# New α- and γ-synuclein immunopathological lesions in human brain

**DOI:** 10.1186/s40478-014-0132-8

**Published:** 2014-09-11

**Authors:** Irina Surgucheva, Kathy L Newell, Jeffrey Burns, Andrei Surguchov

**Affiliations:** Retinal Biology Research Laboratory, VA Medical Center, 4801 East Linwood boulevard, 64128 Kansas City, MO USA; Department of Neurology, Kansas University Medical Center, 3901 Rainbow Boulevard, 66160 Kansas City, KS USA; Alzheimer’s Disease Center, Department of Neurology, Kansas University Medical Center, 3901 Rainbow Boulevard, 66160 Kansas City, KS USA

**Keywords:** Lewy bodies, Alzheimer’s disease, Parkinson’s disease, Alpha-synuclein, Gamma-synuclein

## Abstract

**Introduction:**

Several neurodegenerative diseases are classified as proteopathies as they are associated with the aggregation of misfolded proteins. Synucleinopathies are a group of neurodegenerative disorders associated with abnormal deposition of synucleins. α-Synucleinopathies include Parkinson’s disease, dementia with Lewy bodies, and multiple system atrophy. Recently accumulation of another member of the synuclein family- γ−synuclein in neurodegenerative diseases compelled the introduction of the term γ−synucleinopathy. The formation of aggregates and deposits of γ−synuclein is facilitated after its oxidation at methionine 38 (Met^38^).

**Results:**

Several types of intracytoplasmic inclusions containing post-translationally modified α- and γ−synucleins are detected. Oxidized Met^38^-γ-synuclein forms aberrant inclusions in amygdala and substantia nigra. Double staining revealed colocalization of oxidized-γ-synuclein with α-synuclein in the cytoplasm of neurons. Another type of synuclein positive inclusions in the amygdala of dementia with Lewy bodies patients has the appearance of Lewy bodies. These inclusions are immunoreactive when analyzed with antibodies to α-synuclein phosphorylated on serine 129, as well as with antibodies to oxidized-γ-synuclein. Some of these Lewy bodies have doughnut-like shape with round or elongated shape. The separate immunofluorescent images obtained with individual antibodies specific to oxidized-γ-synuclein and phospho-α-synuclein clearly shows the colocalization of these synuclein isoforms in substantia nigra inclusions. Phospho-α-synuclein is present almost exclusively at the periphery of these structures, whereas oxidized-γ-syn immunoreactivity is also located in the internal parts forming dot-like pattern of staining.

We also identified several types of oxidized-γ-syn positive astrocytes with different morphology and examined their immunohistochemical phenotypes. Some of them are compact cells with short processes, others have longer processes. Oxidized-γ-synuclein positive astrocytes may also display mixed morphological and immunocytochemical phenotypes between protoplasmic and fibrous astrocytes.

**Conclusions:**

These results reveal new γ−synuclein positive lesions in human brain. Oxidized-γ-synuclein is colocalized with phospho-α-synuclein in doughnut-like inclusions. Several types of astrocytes with different morphology are immunopositive for oxidized-γ-synuclein.

## Introduction

The pathophysiological changes associated with neurodegenerative diseases (NDDs) begin decades before the emergence of clinical symptoms. Understanding the early mechanisms associated with NDDs pathology is, therefore, important for identifying disease-modifying therapeutic targets. Recent genetic and biochemical analysis has confirmed that the abnormal accumulation of naturally unfolded proteins in the brain is central to several NDDs. These “cerebral proteopathies” include dementia with Lewy bodies (DLB), Alzheimer’s disease (AD), Parkinson’s disease (PD) and a variety of other disorders. AD, the most common form of dementia, is characterized by accumulation of intraneuronal deposition of the hyperphosphorylated tau protein (neurofibrillary tangles) and extracellular aggregates of β-amyloid (amyloid plaques). However, amyloid accumulation often also occurs in patients with DLB and in some patients with PD with dementia [1]. At the same time more than 50% of AD cases exhibit abundant brain accumulation of α-synuclein (α-syn) positive Lewy bodies (LBs) [2,3]. On the other hand, α-syn accumulates in PD and is considered as a potential biomarker of this disorder.

α-Syn (α-syn) is ubiquitously expressed in brain and is highly enriched in presynaptic nerve terminals, where its main physiological function is the regulation of synaptic levels of monoamine neurotransmitters through modulation of vesicular release. The results of analysis of total α-syn in peripheral blood, plasma and cerebrospinal fluid (CSF) do not confirm that it can be used as a reliable biomarker or predictor of disease progression. However, post-translationally modified forms of α-syn and in particular Ser^129^ phosphorylated isoform (phospho-α-syn) [4] might have potential value as a diagnostic tool [5,6].

Quantification of post-translationally modified forms of these proteins elicits more hope to find a new biomarker useful for diagnostic purposes. The finding and characterization of dependable novel biomarkers is still highly unmet and much needed. Initial findings of new biomarkers using post-mortem brain samples may lead to their subsequent testing with biological fluids, i.e. serum or cerebrospinal fluid and become a basis for the development of new diagnostic tools.

Another member of the synuclein family - γ-synuclein (γ-syn) is also aggregation-prone protein which forms intracellular inclusions. γ-Syn is a component of atypical inclusion bodies in human NDDs [7-11]. Furthermore, elevated expression of γ-syn causes selective damage and loss of discrete populations of neurons and their axons [9,12]. Importantly, polymorphisms in the γ-syn locus have been associated with human diffuse Lewy body disease [13]. Formation of heteromeric complexes containing α- and γ-syn was demonstrated [14]. Our recent results indicate that γ-syn is easily oxidized at Methionine-38 (Met-38) forming oxidized-γ-syn (oxi-γ-syn) and after oxidation it can induce aggregation of α-syn [15]. Moreover, γ-syn can be transmitted between cells [15] and presumably to spread pathology as was shown for α-syn [16-18]. These findings raise a possibility that oxidation and/or aggregation of this protein might be involved in the pathogenesis of some NDDs. This prompted us to investigate if γ-syn pathology is present in patients with AD with LB, DLB and PD.

Naturally unfolded proteins, including synucleins, are often post-translationally modified which may serve as a signal for their sequestration in inclusion bodies [4,19,20]. Previously we have found that γ-syn can be easily oxidized and form aggregates and inclusions in cell cultures [15]. Here we used antibodies specific to unmodified and post-translationally modified α- and γ-syns to reveal immunopathology in brain tissues of patients with NDDs. In particular, we used antibodies to pSer^129^-α-syn, α-syn specific antibody raised against LB and antibody to oxi-Met^38^-γ-syn. We also identified several types of oxi-γ-syn positive astrocytes with different morphology and examined their immunohistochemical phenotypes. Finding of new astrocytes markers which could distinguish different types of astrocytes is important for better understanding of their role in healthy brain and in NDDs.

## Materials and methods

Brain specimens of the amygdala and the substantia nigra were obtained from the brain bank of the University of Kansas Medical Center Alzheimer’s Disease Center. Tissue was requested from pathologically-defined cases with the presence of Lewy bodies and included individuals with AD-related changes, PD, and DLB. A primary diagnosis was defined based on the burden of pathology and neuropathological characteristics.

Paraffin sections were used to retrieve two formalin-fixed, paraffin-embedded blocks, one for the amygdala and the other for the substantia nigra. From each of these paraffin blocks, 7 μm sections were cut and mounted on microscope slides. The formalin-fixed paraffin-embedded brain sections were deparaffinized by standard method. Heat-mediated antigen retrieval procedure using 10 mM sodium citrate buffer, pH-6.0 was applied. Permeabilization was done in PBS containing 0.2% Triton X-100 for 45 min at RT. Blocking was carried out overnight at +4°C in 10% normal horse serum containing 0.05% Triton-X-100.

### Antibodies

For immunofluorescent staining the following primary antibodies were used.

#### Primary antibodies

Polyclonal antirabbit antibody to oxi-γ-syn was generated as described in our previous publication [15], α-syn antibody (ab6162 from Abcam, San Francisco, CA) was generated to human synthetic peptide in sheep, pSer^129^-α-syn from Wako (Richmond, VA), monoclonal antimouse GFAP (Mab360, Millipore, Temecula, CA), polyclonal antigoat - Iba1 - (Cat. #ab 107159, Abcam, San Francisco, CA) have been used for immunofluorescent staining. The following monoclonal antibodies have been also applied: antimouse β-amyloid (clone 6E10, Covance, Princeton, NJ) and antimouse γ-syn antibody (clone 1H10D2, Antagene, Mountain View, CA). Antibodies to the following astrocytes markers have been used: monoclonal anti-Aldh1L1, clone N103/39 (Cat #MABN495, NeuroMab UC, Davis, CA) is used as a protoplasmic astrocytes marker. Monoclonal mouse antihuman CD44, phagocytic glycoprotein-1 (Clone F10-44-2, Dako, Carpentaria, CA) was used to identify CD44+ astrocytes. Mouse anti-glutamine synthetase (Cat# 610517, BD Transduction Laboratories, Franklin Lakes, NJ) used as a general astrocyte marker.

#### Secondary antibodies

The following secondary antibodies were used. Donkey antisheep- IgG-TR (Cat# sc-3913, Santa Cruz, Dallas, Texas), Alexa Fluor 594-goat antimouse, Alexa Fluor 594-donkey antigoat, Alexa Fluor 488 – donkey antirabbit were from Jackson ImmunoResearch Lab (West Grove, PA). Incubation with secondary antibodies was carried out for 2 h at RT in the dark. Autofluorescence Eliminator Reagent (Millipore, Temecula, CA) was applied to reduce autofluorescence. Finally, the slides were covered by Vectashield –mounting medium containing DAPI (Vector Labs, Burlingame, CA). The images were received using a Nikon 80i Upright scope supplied with the digital camera Olympus DP72 (Center Valley, PA) and the software Olympus Cell Sense.

### Immunofluorescence staining

Was performed on 8 μm thick frozen sections. The sections were fixed in 2% paraformaldehyde for 10 min, washed and then permeabilized in 0.2% Triton X-100 in PBS for 1 h. The slides were blocked with 10% normal horse serum in PBS + 0.05% Triton X-100 and then the primary antibodies were added at a dilution 1:100 for overnight incubation at 4°C in 5% normal horse serum in PBS + 0.05% Triton X-100. The following day the slides were washed 3 times (PBS + 0.05% Triton X-100), 15 min each and incubated with secondary fluorescent antibodies (Alexa Fluor conjugates) at 1:500 dilution and Donkey anti-sheep Texas red at 1:250 dilution for 2 h at RT followed by washing as described above. Then the slides were mounted in VectaShield with DAPI (Vector Laboratories, Burlingame CA). The slides were imaged using an Olympus BX46 fluorescent microscope at 40× magnification.

### Immunochemistry

The slides were treated as described above with the following modifications. After blocking the sections were incubated with polyclonal oxi-γ-syn antibody (dilution 1:100) overnight at 4°C. Unbound antibody was removed by washing in PBS and the incubation with rabbit HRP conjugated sustained for 1 h at RT. Then Vectastatin kit was used (Vector Laboratories, Burlingame, CA) and the staining was visualized by precipitation of Diaminobenzidine (DAB) (Sigma Aldrich, St. Louis, MO) following the manufacturer’s instructions. In negative controls the primary antibodies were omitted.

### Calculation of oxi-γ-syn positive astrocytes

For calculation of oxi-γ-syn positive astrocytes we used 7 amygdala and 7 substantia nigra slices for each case. After staining we calculated the number of oxi-γ-syn astrocytes present on the whole slices.

### Western blotting

The brain extracts (30 μg total protein) were analyzed by WB in a 12% polyacrylamide gel (PAAG) in the presence of SDS as described previously [10,15]. After electrophoresis, proteins were transferred onto an Immobilon-FL transfer membrane (0.45 μm, Millipore, Chelmsford, MA). Nonspecific binding sites were blocked by immersing the membrane in PBS with 5% nonfat dry milk for 1 h at room temperature (RT) on an orbital shaker. For quantitative imaging of bands an infrared imaging system (Odyssey, LiCor, Lincoln, NE) was used. Other details have been described previously [15].

## Results

**Figure 1:**
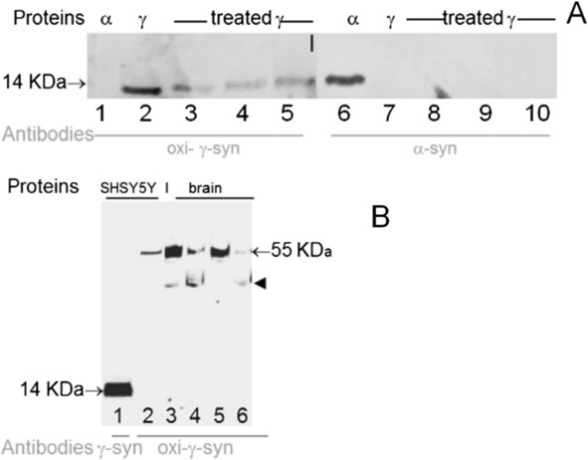
**Specificity of synuclein antibody toward recombinant proteins (A) and extracts from cell culture and brain (B). A**: Recombinant α-syn (lanes 1 and 6), γ-syn (lanes 2 and 7) and γ-syn treated with DA (oxi-γ-syn, lanes 3–5 and 8–10) were separated in 12% polyacrylamide gel with SDSNa and tested with antibodies specific to different synucleins isoforms. The following amounts of protein were subjected to electrophoresis: lanes 2 and 7 – untreated -γ-syn - 62.5 ng. Since in the course of the treatment by 250 μM DA the amount of immunoreactive monomeric γ-syn was reduced, we applied increasing amounts of proteins: 100 ng on lanes 3 and 8; 187.5 ng on lanes 4 and 9; and 225 ng on lanes 5 and 10. WB was probed with antibodies against oxi-γ- syn (lanes 1–5) and antisheep-α-synuclein (lanes 6–10). **B**: Antibody to unmodified γ-syn (ABcam, 55424) recognizes monomeric form of γ-syn (lane 1), while antibody to oxi-γ-syn recognizes tetrameric γ-syn in cell extracts of SH-SY5Y cells overexpressing γ-syn (lane 2) and in extracts of human brain (3–6). Lanes 3 and 5 extracts from amygdala were analyzed; lanes 4 and 6 – extracts from substantia nigra from control individuals.

Since we used home-made antibody to oxi-γ − syn we first tested its specificity using recombinant proteins, extracts from cultured cells and brain extracts. Oxi-γ-syn was prepared by incubation of recombinant protein with 250 μM dopamine (DA) as described previously [15] (marked “treated γ“ on Figure 1, lanes 3–5 and 8–10). Antibody to oxi-γ-syn does not cross react with α-syn (Figure 1A, lane 1) and antibody to α-syn does not cross react with γ-syn (Figure 1A, lanes 7–10). Both these antibodies recognize only corresponding monomeric recombinant protein with molecular weight ~14 KDa (lane 2 for γ − syn and lane 6 for α-syn). However, when used with extracts from cultured cells (Figure 1B, lane 2) or brain extracts (lanes 3–6) antibody to oxi-γ-syn recognizes tetrameric form of γ-syn (~55 Kda, arrow on Figure 1B). In addition to tetramers lower molecular weight bands are present in brain extracts which are presumably products of the tetramer degradation (arrowhead, lanes 3–6, Figure 1B).

**Figure 2:**
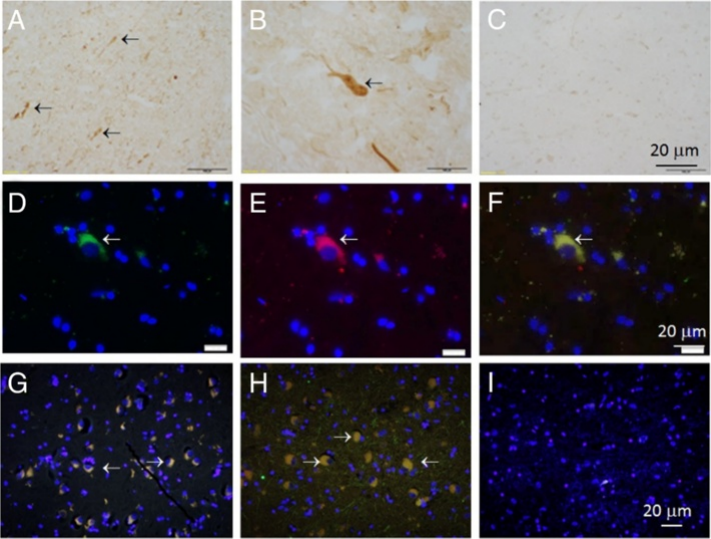
**Colocalization of α-syn with oxi-γ-syn in amygdala and substantia nigra in AD with LB patients.** Frozen (two top rows) and paraffin-embedded sections (bottom row) of substantia nigra and amygdala of healthy **(A, G)** and AD patients with DLB **(H, I)**. The top row: frozen sections **(A and B)** were stained using H&R- DAB –procedure and oxi-γ-syn antibody. **A**- substantia nigra from K46 healthy individual, **B** - substantia nigra from S3 patient (AD with LB), **C**- the same as in **A**, but without primary antibody. In the middle row the sections of substantia nigra from S3 patient were stained by oxi-γ-syn antibody; **D** - α-syn antibody (Abcam ab6162, antisheep); **E** - oxi-γ-syn antibody; **F** - colocalization of both antibodies. The bottom row. The sections were double stained with antibody against α-syn (red, Abcam ab 6162, antisheep) and oxi-γ-syn (green, antirabbit, homemade antibody). Yellow cytoplasmic staining is shown by an arrow in a sample from amygdala of K21-healthy donor **(G)** and in a sample from amygdala taken from a patient S4 with AD with LB **(H)** substantia nigra from patient S3 suffering from mild AD with LB **(I)**. Antibody to unmodified γ-syn (clone 1H10D2, Antagene) (red) reveals a weak staining of unmodified γ-syn in the cytoplasm of neurons.

Then we investigated synuclein localization in brain samples from patients and controls using antibodies to several synuclein isoforms. Accumulation of aberrant oxi-γ-syn in frozen sections of substantia nigra was revealed by H@R – DAB procedure in neurites (Figure 2A, control individual, case K46) and cytoplasm (B, DLB patient, case S3). Since oxi-γ-syn may initiate α-syn aggregation [15] we examined whether these synuclein isoforms have overlapping intracellular localization. α-Syn (D) and oxi-γ-syn (E) are present in overlapping cytoplasmic localization (a merged image F) in frozen sections of substantia nigra. A similar overlapping cytoplasmic localization was observed when formalin–fixed paraffin-embedded slices were used for double staining (G-I). Oxi-γ-syn positive cells were identified as neurons with neuronal marker MAP2 (not shown).

**Figure 3:**
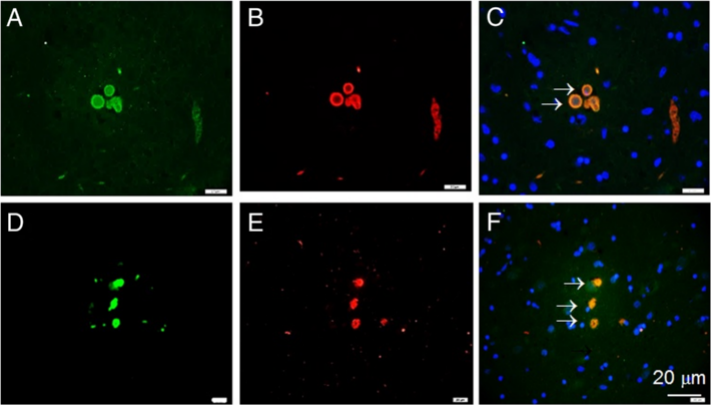
**The presence of post-translationally modified α- and γ-syn in LB.** The immunofluorescence staining of sections by individual antibody. **A-C** - substantia nigra of S3 patient (AD with LB). **A** - oxi-γ-syn immunoreactivity, **B**- phospho-α-syn immunoreactivity, **C**- merged. **D**-**F** -LB from amygdala of a patient K33 with DLB. **D** - oxi-γ-syn immunoreactivity, **E**- phospho-α-syn immunoreactivity, **F** – merged. Blue - DAPI staining.

Intracytoplasmic inclusions with appearance of LB were detected in the amygdala of DLB patients immunoreactive with an antibody specific to phosphorylated on Ser^129^ α-syn, as well as with antibody to oxi-γ-syn (Figure 3). Some of these structures have doughnut-like shape with round (A- C) or elongated (D-F) morphology; they are identified in substantia nigra by double staining with an antibody specific to phosphorylated Ser^129^ α-syn (B, E) and antibody to oxi-γ-syn (A,D). Colocalization of phospho-α**-**syn (red) and oxi-γ-syn (green) in LB-like structures is shown in Figure 3C and F. In these structures α-syn staining is present almost exclusively as peripheral ring-like pattern (Figure 3B), whereas oxi-γ-syn immunoreactivity is also located in the internal parts forming dot-like pattern of staining (Figure 3A).

**Figure 4:**
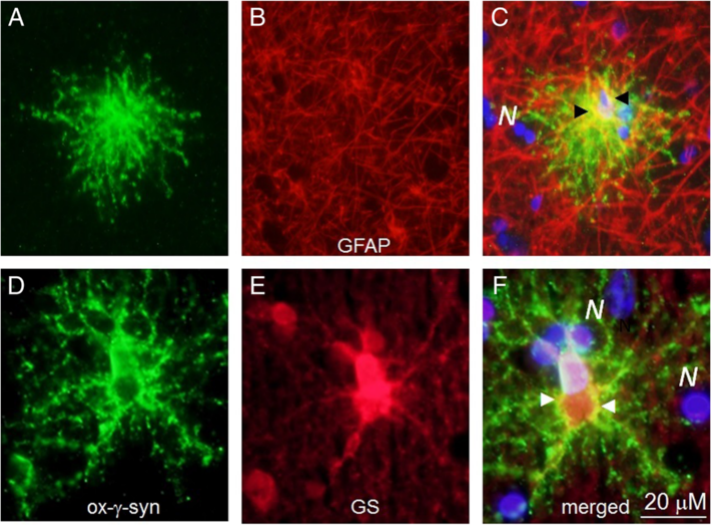
**Oxi-γ-syn positive cells with long processes are identified as astrocytes with specific markers, GFAP (A-C) and glutamine synthetase (D- F).** GS-positive astrocytes have relatively long branched processes **(D)** compared to more compact bushy-like spherical GFAP-positive astrocytes **(A)**.

**Figure 5:**
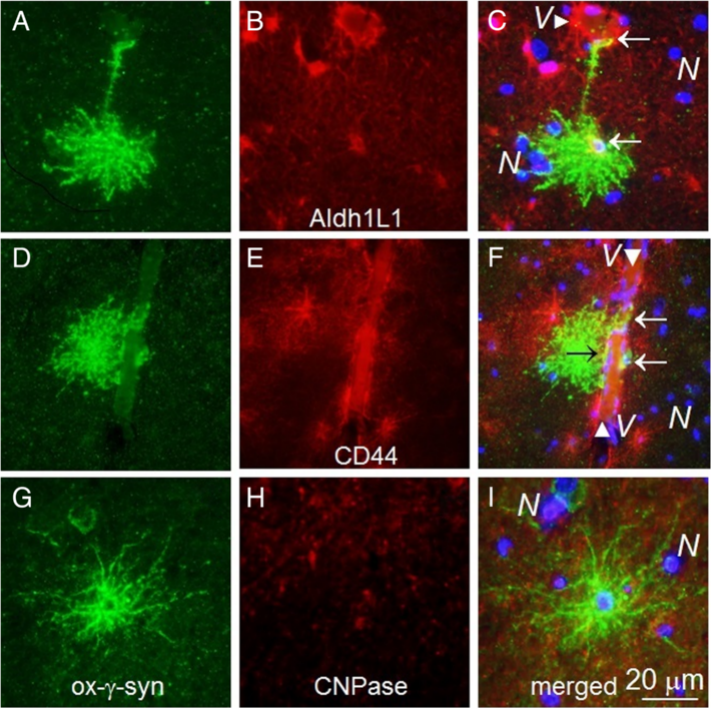
**Oxi-γ-syn positive astrocytes have a partial colocalization with astrocyte markers Aldh1L1 (C) and CD44 (F) (arrows) and are not colocalized with an oligodendrocyte marker CNPase (I). A**, **D**, **G** – oxi-γ-syn staining. **B** – Aldh1L1, **E**- CD44, **H** –CNPase staining. Some of oxi-γ-syn-positive astrocytes reside near large blood vessels **(** ***V,*** **C and F)** with processes extending toward the vessel surface **(C)**.

In addition to the presence in lesions described above oxi-γ-syn is localized in several types of astrocytes, which are identified by colocalization with astrocyte markers GFAP (Figure 4, A, C) and glutamine synthetase (D and F). A partial colocalization of oxi-γ-syn immunoreactivity with other astrocyte markers, Aldh1L1 (Figure 5A, C) and CD44 (D, F) is shown on Figure 5. Oxi-γ-syn-positive astrocytes are phenotypically heterogeneous, having different shape and length of their processes (Figures 4 and 5, green staining). They are identified as astrocytes using GFAP antibody (Figure 4, C) and glutamine synthetase (GS) antibody (Figure 4, F). GS-positive astrocytes have relatively long branched processes (Figure 4, D-F) compared to more compact bushy-like GFAP-positive astrocytes (Figure 4, A, C). Some types of oxi-γ-syn positive astrocytes have a partial overlap with such astrocytes markers, as Aldh1L1 (Figure 5C) and CD44 (Figure 5, F), while there is no overlap with an oligodendrocyte marker CNPase (Figure 5I). Some of oxi-γ-syn-positive astrocytes reside near large blood vessels (*V*) (Figure 5C and F) with processes extending toward the vessel surface (Figure 5C). The number of oxi-γ-syn–positive astrocytes is the highest in PD patients and differs significantly in patients and controls (Table 1).

**Table 1:** **The amount of oxi- Met** ^**3**^ **- γ-synuclein –positive astrocytes in patients with NDDs and control individuals**

**Patient’s ID**	**Sex**	**Age**	**Diagnosis**	**Number of oxi-Met** ^**38**^ **-γ-syn–positive astrocytes**
K33	M	76	DLB	+
K11	F	87	AD with DLB related pathology	+
K13	M	85	DLB	++
K39	M	74	DLB	+
S3	M	73	DLB + mild AD	+++
S4	F	87	AD with DLB related pathology	++
PD1	M	55	PD	++
PD2	M	85	PD	++++
K21	M	97	Control	++
K35	F	61	Control	+
K41	F	61	Control	++
K45	M	66	Control	+
K46	M	79	Control	+++
K48	F	88	Control	+++

The patients were selected based on the neuropathological characteristics. The number of oxi-Met^38^ –positive astrocytes was calculated on a 2 x 2.5 cm slice.

+ represents the number of oxi-Met^38^ –positive astrocytes from 1 to 2.

++ represent the number of oxi-Met^38^ –positive astrocytes from 2 to 5.

+++ represent the number of oxi-Met38 –positive astrocytes from 5 to10.

++++ represent the number of oxi-Met38 –positive astrocytes > 10.

## Discussion

PTMs have emerged as important determinants of protein physiological and pathological functions. Several PTMs are enriched within LB and exist at higher levels in α-synucleinopathy brains. Synuclein’s PTMs interfere with its aggregation propensity and cellular signaling [19-22]. These findings suggest that PTMs of synucleins may signal a shift to a toxic gain of function and/or sequestration into insoluble structures. Here we have shown that phospho-α-syn and oxi-γ-syn are colocalized in LB in amygdala and substantia nigra of DLB, PD and some AD patients. The results of Western blotting show the absence of cross reactivity between antibodies we used for identification of α- and γ-syn. We also revealed another type of γ-syn immunopositive staining of astrocytes which are present both in DLB patients and in control brain samples. The presence of oxi-γ-syn in intracellular deposits may have an important role in the process of inclusion formation, since oxi-γ-syn induces protein aggregation [15] and thus may initiate protein deposition.

Interestingly, intracellular inclusions similar to doughnut-like LB shown on Figure 2B-D have been described recently in DLB patients using antibodies to α-syn and caspase-cleaved TDP-43 [23]. Both markers are uniformly distributed in these structures in PD cases, but peripherally distributed in patients with DLB.

In contrast to formation of synuclein inclusions in neurons, we did not find similar inclusions and deposits in astrocytes, although glial accumulation of α-syn was described previously, especially at the step of initiation of NDDs [24,25]. The role of neuron-glia interaction in the etiology of NDDs is an important area of current investigations due to the involvement of naturally unfolded proteins in prion-like transmission of pathology [16-18,24]. The results presented here are in agreement with previous observations of aberrant γ-syn accumulation in the optic nerve astrocytes and spheroids which is not accompanied by the formation of inclusion bodies or deposits [10,11]

We identified several types of oxi-γ-syn positive astrocytes with different morphology and examined their immunohistochemical phenotypes. Some of them are compact with short processes (Figure 4A and D) looking similar to protoplasmic astrocytes [26,27], others have longer processes (Figure 4D and G) which may end on a blood vessel (Figure 5C). Some of the oxi-γ-syn positive astrocytes display mixed morphological and immunocytochemical phenotypes between protoplasmic and fibrous astrocytes (Figure 4A and C). The quantity of oxi-γ-syn positive astrocytes differs significantly in patients with different NDDs and controls. They are most frequent in patients with PD (Table 1). Astrocytic α-syn is considered an important component of the pathology [28,29], however the role of γ-syn and post-translationally modified forms of synucleins in neurodegeneration is not completely understood.

The results presented here might be important for better understanding of mechanisms initiating the formation of protein deposits in NDDs. Findings from several recent studies have suggested that aggregation-prone proteins, such as tau, α-syn, polyglutamine-containing proteins, and amyloid-β, can enhance each other’s aggregation [30-35]. In particular, β-amyloid augments α-syn accumulation [33,34], Tau increases α-syn aggregation [31,32] while synergistic interactions between β-amyloid, tau, and α-syn might accelerate neuropathology [33]. Importantly, mechanism of hybrid oligomer formation plays an essential role in the pathogenesis of combined AD and PD [30].

A role of γ-syn and its post-translationally modified forms in NDDs are not investigated in such details as of α-syn and other naturally unfolded proteins. However, neuronal accumulation of aberrant γ-syn in NDDs suggests that γ-syn plays an important, yet not completely understood role in these disorders. Furthermore, its presence in cerebrospinal fluid and elevation in aged subjects with neurodegenerative and vascular changes suggest that γ-syn can be used as a biomarker of neurodegeneration, gliosis in dementia with Lewy bodies and other NDDs [36].

Recent studies have demonstrated that α- and γ-syn interact with each other and these interactions increase the propensity of synucleins to aggregate [15,37]. Co-immunoprecipitation experiments showed heteromeric protein: protein complexes that included both α- and γ-syns [14,15]. Thus, antibody to oxi-γ-syn can be used as a new biomarker for NDDs both individually and in combination to antibodies to other markers of neurodegeneration. The presence of both α-syn and γ-syn in brain deposits in several NDDs may suggest a common element in the mechanism leading to these disorders.

Astrocytes exert many essential and complex functions and react to all forms of CNS insults, including NDDs with a response referred to as reactive astrogliosis. Finding of new astrocytes markers which could differentiate various types of astrocytes is important for better understanding of their role in healthy brain and in NDDs.

## Conclusions

The results presented here confirm previous findings that accumulation of γ-syn may occur in human brain. Lesions immunopositive for both α-syn and γ-syn, as well oxi-γ-syn positive astrocytes with different morphology are described.

## Abbreviations

AD: Alzheimer’s disease
DLB: Dementia with Lewy body
LB: Lewy body
NDDs: Neurodegenerative diseases
PD: Parkinson’s disease
Phospho-α-syn: Phosphorylated at Ser-129 α-synuclein
Oxi-γ-syn: Oxidized at Met-38 γ-synuclein
